# Diversity of synaptic protein complexes as a function of the abundance of their constituent proteins: A modeling approach

**DOI:** 10.1371/journal.pcbi.1009758

**Published:** 2022-01-18

**Authors:** Marcell Miski, Bence Márk Keömley-Horváth, Dorina Rákóczi Megyeriné, Attila Csikász-Nagy, Zoltán Gáspári

**Affiliations:** 1 Faculty of Information Technology and Bionics, Pázmány Péter Catholic University, Budapest, Hungary; 2 Cytocast Ltd., Vecsés, Hungary; 3 Randall Centre for Cell and Molecular Biophysics, King’s College London, London, United Kingdom; National Research Council, ITALY

## Abstract

The postsynaptic density (PSD) is a dense protein network playing a key role in information processing during learning and memory, and is also indicated in a number of neurological disorders. Efforts to characterize its detailed molecular organization are encumbered by the large variability of the abundance of its constituent proteins both spatially, in different brain areas, and temporally, during development, circadian rhythm, and also in response to various stimuli. In this study we ran large-scale stochastic simulations of protein binding events to predict the presence and distribution of PSD complexes. We simulated the interactions of seven major PSD proteins (NMDAR, AMPAR, PSD-95, SynGAP, GKAP, Shank3, Homer1) based on previously published, experimentally determined protein abundance data from 22 different brain areas and 42 patients (altogether 524 different simulations). Our results demonstrate that the relative ratio of the emerging protein complexes can be sensitive to even subtle changes in protein abundances and thus explicit simulations are invaluable to understand the relationships between protein availability and complex formation. Our observations are compatible with a scenario where larger supercomplexes are formed from available smaller binary and ternary associations of PSD proteins. Specifically, Homer1 and Shank3 self-association reactions substantially promote the emergence of very large protein complexes. The described simulations represent a first approximation to assess PSD complex abundance, and as such, use significant simplifications. Therefore, their direct biological relevance might be limited but we believe that the major qualitative findings can contribute to the understanding of the molecular features of the postsynapse.

## Author summary

Chemical and electrical synapses connect neurons in the brain. In chemical synapses the information is sent via molecules from one neuron (presynaptic one) to the other neuron (postsynaptic one). The messenger molecule called neurotransmitter is released from the presynaptic neuron’s active zone and binds to receptor molecules sitting on the postsynaptic neuron’s cell surface. This part of the postsynaptic neuron is the dendrite. Inside the dendrite there is an electron dense region full of proteins binding to each other forming large protein complexes. These complexes make sure that the receptor molecules are on the right place usually in front of the active zone. The protein dense region of the postsynaptic cell in the dendrites is called the postsynaptic density. We have performed extensive simulations on the formation of postsynaptic protein complexes using a well-defined set of proteins and a large number of publicly available input data sets on protein abundance. We used a simulator implementing the Gillespie algorithm to simulate binding and unbinding events proteins. We found that the relationship between single protein and protein complex abundances can be non-trivial, since similar complex distributions can emerge from distinct relative protein abundances and quite different protein complexes can be formed from almost similar initial protein abundances. Our results are compatible with the idea that the association-dissociation of smaller subcomplexes lead to the formation of large supercomplexes. The emergence of supercomplexes is largely facilitated by the self-association of Homer1 and Shank3 proteins. Our results are qualitatively in agreement with the formation of the experimentally observed ‘nanodomains’ in the postsynaptic density.

## Introduction

Complexity of the human brain is often attributed to the diversity of the neuronal network. However, there is growing experimental evidence showing that individual synapses are highly diverse in terms of the relative abundance of their constituent proteins [1]. These observations have led to the formulation of the synaptomic theory [2] that emphasises the genetic background and experience-dependent changes in the molecular composition of synapses. Notably, identification of the pre- and postsynaptic proteomes is still ongoing with the postsynaptic one estimated to be nearer to saturation [3]. The postsynaptic density (PSD) is an intricate network of proteins located at the postsynaptic membrane and is responsible for signal processing. Due to its complexity, the structure of the PSD is still elusive as a whole. Understanding the organization of the protein network is key to describe physiological and pathological molecular processes underlying learning, memory and behavior.

As demonstrated recently, network-based analysis of the synaptic proteome is a powerful tool and can suggest novel associations between diseases [3]. To get a deeper understanding into the actual mechanism of the PSD, we need to move toward explicit modeling of the protein complexes formed. Our recent sequence analysis [4] suggests that PSD proteins are particularly enriched in domains and regions mediating protein-protein interactions, resulting in a high diversity of possible interactions. Thus, many different complexes and networks can be built from the very same elements depending on their abundance and availability. This view is consistent with the observations that different sets of PSD proteins are capable of forming phase-separated condensates [5, 6] In vivo investigations indicate the presence of supercomplexes and nanodomains involved in the clustering of membrane receptors [7, 8]. The formation of these nanodomains is also dictated by the expression pattern of specific scaffold proteins [7].

While knock-out experiments can provide valuable information on the role of a given PSD protein at the level of the full brain/organism, like the contribution of PSD-95 to learning processes [9], the mechanistic mode of action is only accessible when information about the functional protein interactions and complexes is available. Due to the complexity of the PSD, this kind of data is not readily accessible. In general, experimental methods for global characterization of protein complexes include combinations of gel electrophoresis and size exclusion chromatography with quantitative mass spectrometry, requiring thorough computational analysis [10]. However, current experimental methods can only provide limited information about such a complex and dynamic system like the postsynaptic density. In contrast, the abundance of individual proteins can be measured under cellular conditions [11] and such data sets are available [12]. Using the abundance data and the possible interactions between proteins, the distribution of possible supramolecular complexes can be modeled using a systems biology approach [13].

The Simulation based Complex Prediction (SiComPre) method has been proposed to predict compositions and abundances of protein complexes from the abundance of individual proteins [14]. The method is based on the proteome-wide simulation of protein-protein interactions by the Gillespie algorithm [15]. This algorithm has been widely used for the simulation of various phenomena in the area of proteomics. Research groups using the Gillespie algorithm address questions that are difficult or outright impossible to be answered experimentally with the available methodologies. One of these questions is the case of co-translational protein folding predicting folding/unfolding and codon translation rates [16]. Stochasticity in gene expression regulatory pathways was also studied using the Gillespie algorithm [17], as well as protein degradation by the proteasome [18].

SiComPre has been used to predict the formation of protein complexes in yeast and human cells [14], and changes in the complexome upon drug treatments [19]. Based on the core ideas behind SiComPre a novel whole cell simulation platform was developed, under the name of Cytocast Cell Simulator (www.cytocast.com). This tool is available upon licensing or collaboration with the developer company. The performance of SiComPre was analyzed in detail and it was shown that it already overcomes several limitations of current methods predicting protein complexes by protein-protein interactions and simulations [13].

In the present work we have used Cytocast Cell Simulator to model protein complex formation in the PSD using published mRNA abundance data in 22 different brain areas from a 42 human individuals, totaling 524 sets overall (not all areas are represented for all subjects) [20]. The data sets were obtained from brainspan.org (sets denoted RNA-Seq Gencode v10 summarized to genes) and mRNA abundances have been converted to protein abundances by assuming linear relationship between mRNA and protein amounts (see Materials and methods). We have chosen this data source because it covers a large number of different brain areas and patients, providing a highly versatile data set especially suitable for our investigations. It is clear that our results cannot accurately capture *in vivo* PSD complexes because only a small subset of PSD proteins and their interactions are considered. Nonetheless, we clearly demonstrate the added value of protein complex modeling in the interpretation of protein abundance data and the new biological insights it brings. A custom simulation with any combination of the simulated protein abundances can be run through our server available at psdcomplexsim.cytocast.com.

### PSD proteins investigated

In this study we investigated a small subset of the most well-characterized PSD proteins. Evidently, even the diversity of these can not be captured in our simulations as all of these proteins have multiple isoforms with different partner binding properties, while we have considered only the representative ones as defined in UniProt. Moreover, all these proteins have additional binding partners, some of which might not even be characterized yet. Nevertheless, we believe that this subset is a suitable candidate for a first simulation study as described here. Below we briefly introduce the molecules selected for our protein complex simulations.

The NMDA receptor NMDAR was recognized as a coincidence detector by Donald Hebb in 1949 as it has voltage-dependent *Mg*^2+^ binding sites blocking the cation channel in the absence of depolarization. The receptor is one of the most important components affecting Long Term Potentiation through its *Ca*^2+^ permeability and second messenger pathways [21]. It is also required for the effective elimination of unused synapses in the second phase of synaptogenesis [22].

The AMPA receptor AMPAR is also an abundant ionotropic glutamate receptor having both similar and different subunits compared to NMDAR, resulting in different *Ca*^2+^ permeability and the absence of voltage-dependent behavior. Both NMDAR and AMPAR are required for normal functioning of glutamaterg synapses.

PSD-95 is a member of the MAGUK (membrane-associated guanylate kinase) family and is the most abundant scaffold protein in the postsynaptic density. It contains 3 PDZ domains as well as SH3 and a GK domain, all of which mediate protein-protein interactions. The domains PDZ1–2 and PDZ3-SH3-GK are considered to form two supramodules in which the conformational changes occurring in one domain upon ligand binding affect the behavior of neighboring domains [23, 24]. Among others, the GK domain can associate with the protein GKAP and the PDZ domains can mediate interactions with membrane receptors (see below).

SynGAP is a Ras GTP-ase activating protein capable of interacting with PSD-95 with its C-terminal segments [25]. It has specific activity towards the small GTPase Rap. It has a PH, C2 and a GAP domain as well as a coiled coil region mediating homotrimerization [26]. This feature was not explicitly included in our present models.

The GKAP (DLGAP1) protein is almost completely intrinsically disordered. It contains multiple binding sites for the GK domain and DYNLL and has a helical GH1 domain near its C-terminus. [27] Its C-terminal segment can interact with the PDZ domain of Shank3. In our set of seven proteins, GKAP can be considered the link between the layer of the receptors, potentially bound together by PSD-95, and the deeper scaffold proteins Shank3 and Homer1 that are capable of homooligomerization [28].

Shank3 is a member of the Shank (SH3 and multiple anykrin repeat domains protein) family, containing an ankyrin repeat region, and SH3, a PDZ and a SAM domain, as well as a proline-rich segment [29]. Shank3 is well known for its role in autism spectrum disorder, and its overexpression is associated with ADHD and synaptic dysfunction [30, 31]. Its PDZ domain can bind the C-terminus of GKAP and the Pro-rich region is recognized by the EVH1 domain of Homer1 [32]. The SAM domain is capable of self-association [33].

Homer1 contains an N-terminal EVH1 domain and a long coiled-coil segment which mediates homodimerization and also tetramerization of Homer1 molecules. The EVH1 domain can bind proline-rich segments on Shank proteins, metabotropic glutamate receptors as well as IP3 and ryanodine receptors [34]. The Homer1a isoform lacks the coiled coil region and plays a role in PSD reorganization during sleep [35].

These proteins span the entire PSD from the membrane receptors and establish connections with the cytoskeleton and the inner membrane system of the neurons.

## Results

**Fig 1:**
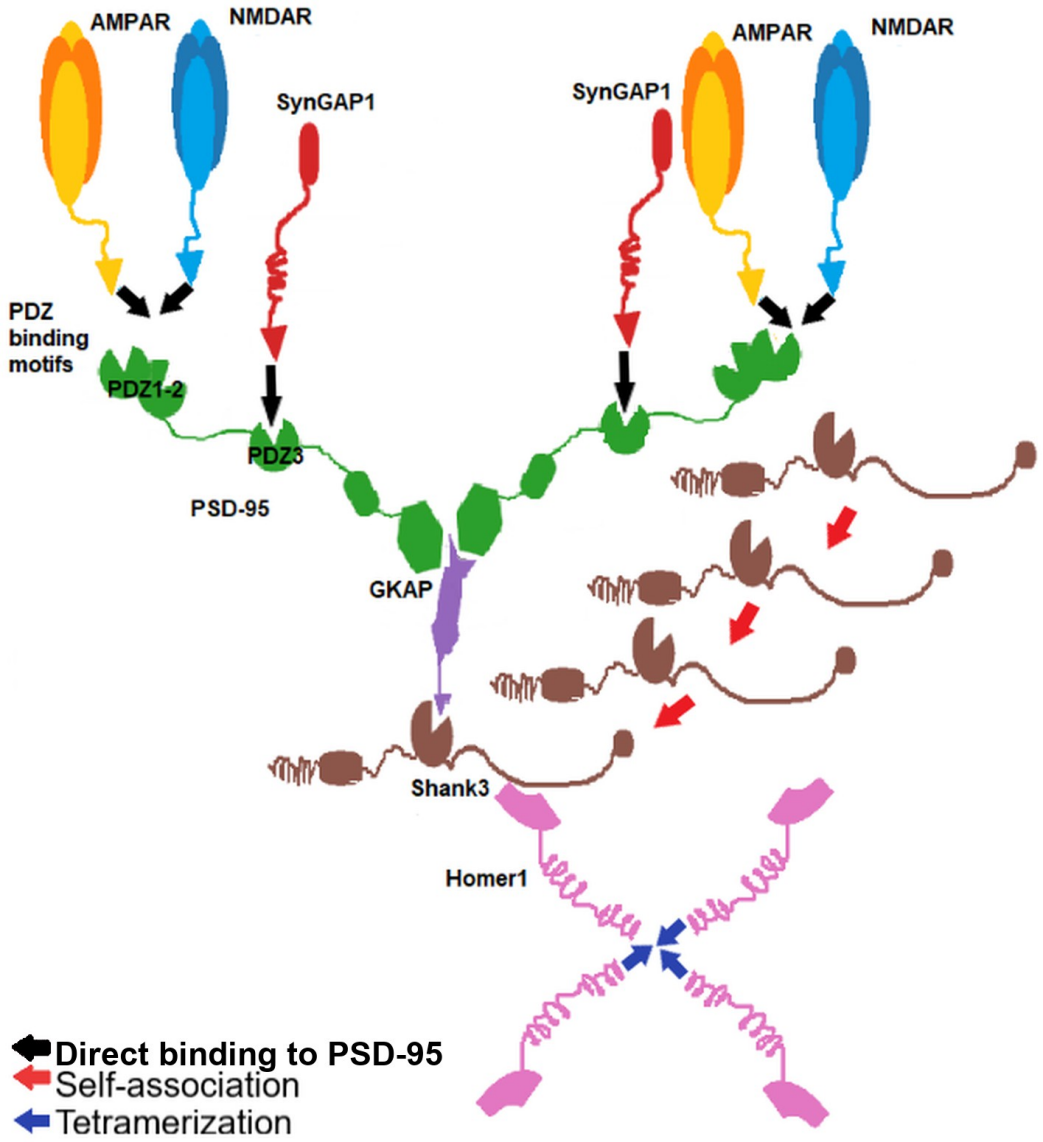
The proteins and their interactions used in this study. The lines represent the interactions considered. The red loop indicates self-association of Shank3 while the blue one refers to the tetramerization of Homer1.

For the present study we chose a well-defined and well-described subset of PSD proteins (Fig 1 and Table A in S1 Table). The seven proteins in this network are the most abundant proteins in the PSD and have well-characterized domain-domain interactions needed for our simulation. They also link the membrane receptors to the actin cytoskeleton, spanning all layers of the PSD, and are also involved in phase separation phenomena [5, 6, 36].

To account for the effect of protein homomultimerization, we have performed simulations with three different setups with regard to protein self-association: in the set designated ‘H4SM’, we have considered Homer1 tetramerization through its coiled coil region and Shank3 polymerization *via* its SAM domain. In the ‘H4’ set we have only considered Homer1 tetramerization, and in the ‘Simple’ settings neither Homer1 tetramerization nor Shank3 self-association was included. Below, unless explicitly noted otherwise, we report the results of the H4SM set as the one with closest to our current understanding of the intracellular behavior of these proteins.

**Fig 2:**
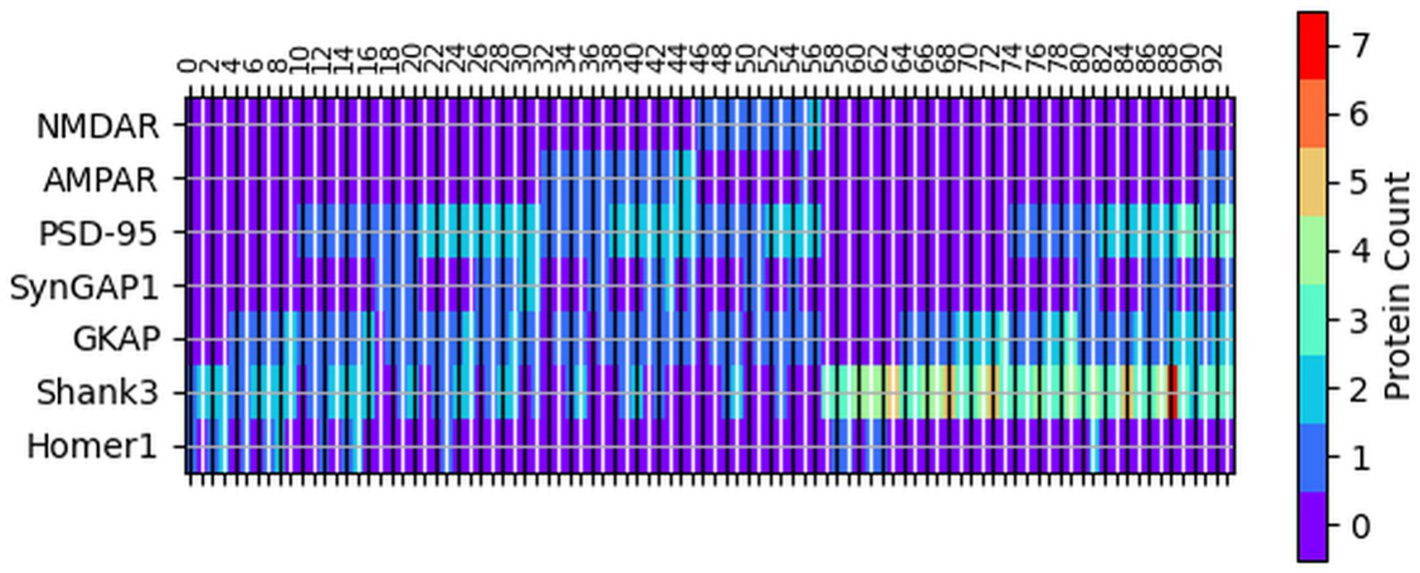
Protein composition of each complex (see text for details) observed in our simulations. The black vertical lines indicate the complexes represented by even ID, the ID of them are shown while the complexes represented by odd ID are indicated by white vertical lines—their IDs are not shown.

To analyze the output of the simulations, we have assigned an ID to each resulting protein complex (Fig 2). Note that here the simplest complexes are shown, i.e. complexes containing the same proteins in the same ratio are treated as a single entity. In other words, in these complexes, referred to as primary complexes below, the numbers of the different components do not have a common divisor larger than 1. This property means that larger associations arising for instance through Homer1 tetramerization and Shank3 multimerization of the same smaller complexes are not considered separately here.

**Fig 3:**
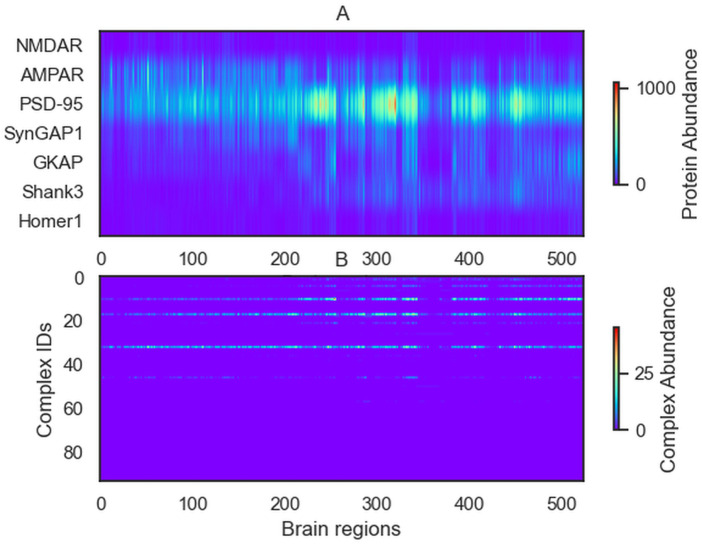
Abundance of each complex obtained in the simulations for each brain region. (A) Input data: abundance of proteins in each brain region. (B) Outputs: Abundance of the complexes. The three most frequent complexes are the PSD-95/GKAP, AMPAR/PSD-95 and the PSD-95/SynGAP dimers.

Abundance of the complexes formed in each simulation are shown in Fig 3. The list of each brain region with IDs are in the Appendix Table A in S1 Table.

### Protein complex distributions are highly sensitive to changes of protein abundances

**Fig 4:**
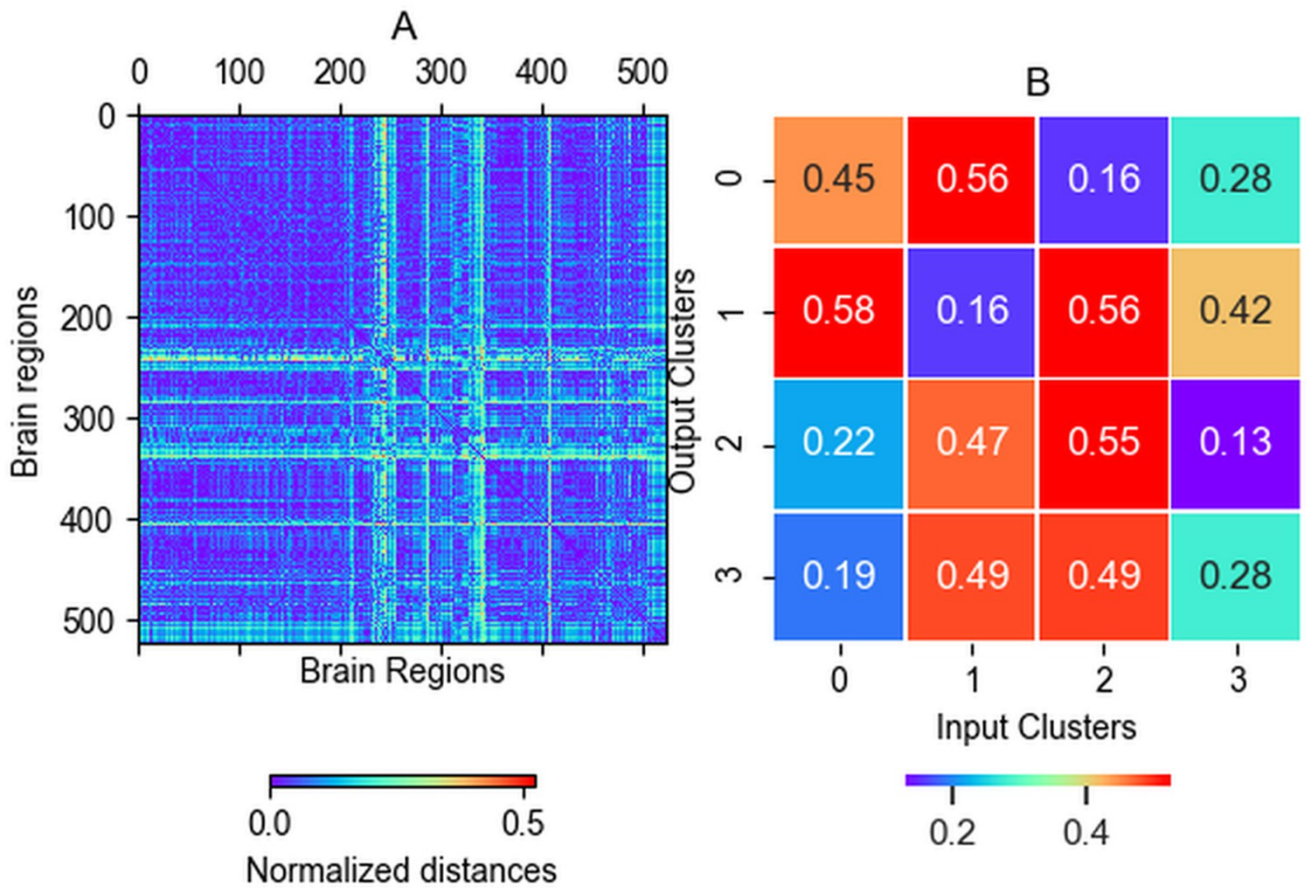
Approaches to identify regions with different characteristics. (A) The difference between cross-region distances. Where the difference is nonzero the outputs of the two regions got closer or farther to each other compared to their distance based on the input data. This value indicates that our simulations provide important additional insights into complex formation. The largest difference is observable at the brain region 238, (H376.IX.51_MFC). There are remarkable differences in regions 340 (H376.VIII.51_STC) and 286 (H376.VI.50_V1C). (B). Cross-distances of clusters based on the input and output data. The result of 4-means clustering is shown. For example, input cluster 0 is closest to output cluster 3, but the difference is 19 percent.

We have analyzed the diversity of input protein abundances and the resulting complexes. It should be noted that in the input there are only seven kinds of proteins with relatively large copy numbers and in the output there are a high number of possible complexes but with much lower copy numbers than the input constituent proteins, precluding direct comparison of their diversity. Both the simulation inputs and outputs can be described by appropriate vectors containing the protein (input) and complex (output) abundance data. We have independently clustered both the input and output vectors and analyzed whether the obtained clusters represent the same sets of experiments. Surprisingly, the input and output clusters show only limited correspondence to each other (Fig 4), indicating that there is a non-trivial relationship between protein abundance and complex occurrence. The difference between input and output clusters was observed for different clustering setups (Fig A in S1 Text).

Besides the differences in the normalized distance matrices, we have calculated the differences between the inputs and outputs based on their first two principal components. The cosine of the angle between the points shows exactly how the relative positions of the two points have changed, not counting the distance. Comparing each region with each region, we find that the heatmap of the distances and the heatmap of the cosines on show a similar pattern. Where the change in distance is greater, the cosine is smaller. Nonetheless, there may be nuanced differences due to the different sensitivities of the measures created. See Fig D in S1 Text. The dominant proteins in the first and second principal components are PSD-95 and AMPAR, respectively.

The most dominant complexes are the binary complexes SynGAP1/PSD-95 (id:75) and AMPAR/PSD-95. These observations resonate with the key role of PSD-95 in the synaptomic theory [2].

**Fig 5:**
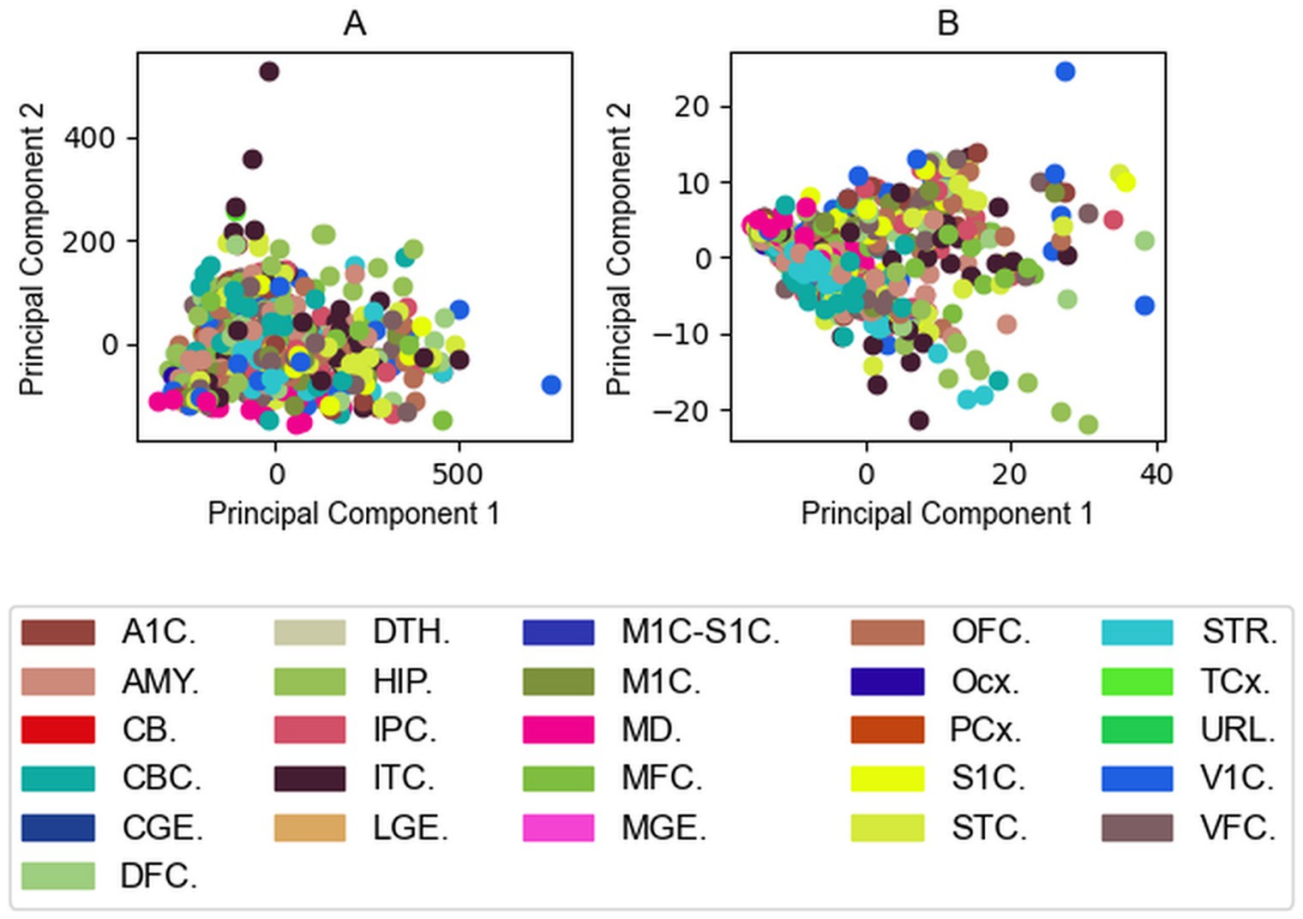
Principal component analyses indicates no significant separation based on the type of brain regions. (A) Brain Regions visualized by the input protein abundance data on the plane of the first two principal components. (B) Brain regions visualized by the output protein complex occurrences on the plane of the first two principal components. The first principal component is mainly affected by PSD-95, the second principal component is mainly affected by AMPAR on the Input Field. The fraction of the variance covered by the first two principal components are 0.69 and 0.16, respectively. Both components are mainly affected by the complex PSD-95/GKAP (id: 10) on the Output Field. The first two principal components cover 0.65 and 0.21 of the variance. The second most affecting complexes are the PSD-95/SynGAP1 (id:17) for the first principal component and GKAP/Shank3 (id:4) for the second component. Not all ITC and V1C-derived data series are spectacularly different from the others, however, one of the sets from both brain regions is still further away in terms of input data. No significant separation is considered in terms of region types—no similar coloured dots appeared separately from other colours. In the input data there is one outlier subject where the abundance of PSD-95 was higher compared to other subjects in almost all brain regions.

Principal component analysis [37] corroborated our assumption that regions move away from each other meaning our simulations provide additional information not trivially deducible from the input abundance data. However, PCA results do not support that the same type of brain regions such as the anterior medial prefrontal cortex would behave similarly during the simulations. In some cases there are some regions of the same type getting closer, but this case is not true in general and no clear trends can be observed (Fig 5). The Fig E in S1 Text shows the distribution of the data from each brain region along PC1. The same qualitative picture can be observed when using all mRNA abundance data, showing that our observation that PCA does not separate brain regions is not attributable to using only data on the seven selected PSD proteins.

**Fig 6:**
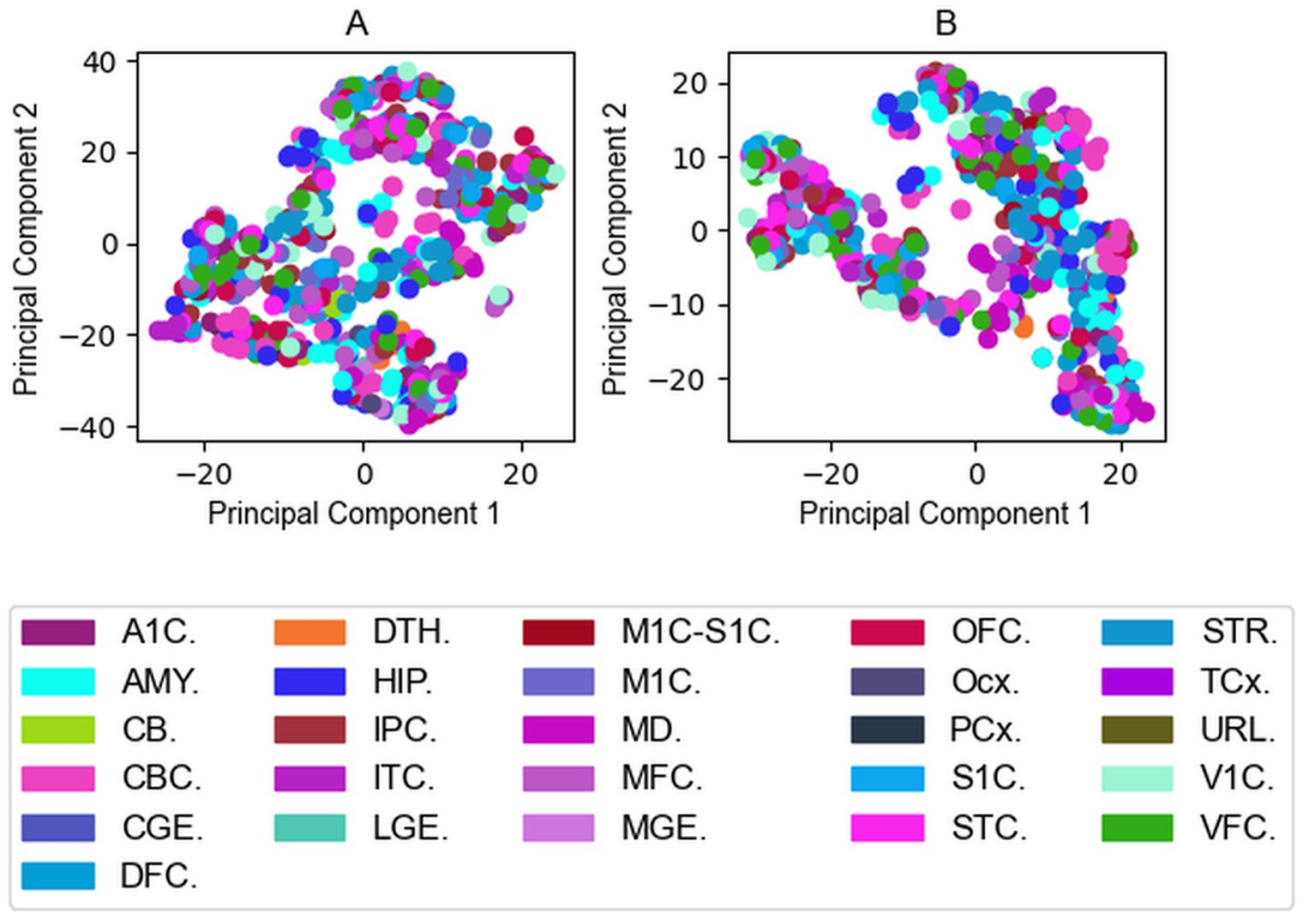
Output of tSNE separates the regions better than PCA. (A) Brain Regions visualized by the input data mapped on a plane by tSNE. (B) Brain regions visualized by the outputs mapped on a plane by tSNE.

In order to complement the PCA results we also run an another dimension reduction method called tSNE based on joint probabilities through input and output data. tSNE separates the regions better but it is still not capable of meaningfully reproduce the regions (Fig 6).

To analyze this phenomenon in more detail, we compared the input and output vectors of each individual experiment by generating two normalized distance matrices. One is based on the input data and the other on the output complex abundance. To assess how the output complex occurrence of a given experiment deviates from the expectations based on its input protein abundance, we subtracted the two distance matrices and identified the largest differences (Fig 4).

We have selected the three experiments with the highest differences in their input and output and discuss these in detail below.

#### Region 238: Anterior medial prefrontal cortex with high abundance of PSD-95

The highest difference in the output relative to the input was observed for brain region 238 (H376.IX.51_MFC), representing data from the anterior medial prefrontal cortex. In this region PSD-95 makes up more than 75% of all proteins, which is unusual as it rarely exceeds 55%. This huge ratio of PSD-95 is subject-specific. The subject H376.IX.51 has similar amount of PSD-95 around 75% in its every region, but it is not disclosed in the data source whether there is any neural disease diagnosed for this individual ([20]).

Considering the receptors, the abundance of AMPAR is much higher that that of NMDAR, which is negligible. Shank3 is the second-most abundant protein while GKAP is in only present in small amounts. The output complexes are dominated by PSD-95/GKAP,PSD-95/SYNGAP and AMPAR/PSD-95 binary interactions. Shank3 is mainly found in Shank3 dimers.

**Fig 7:**
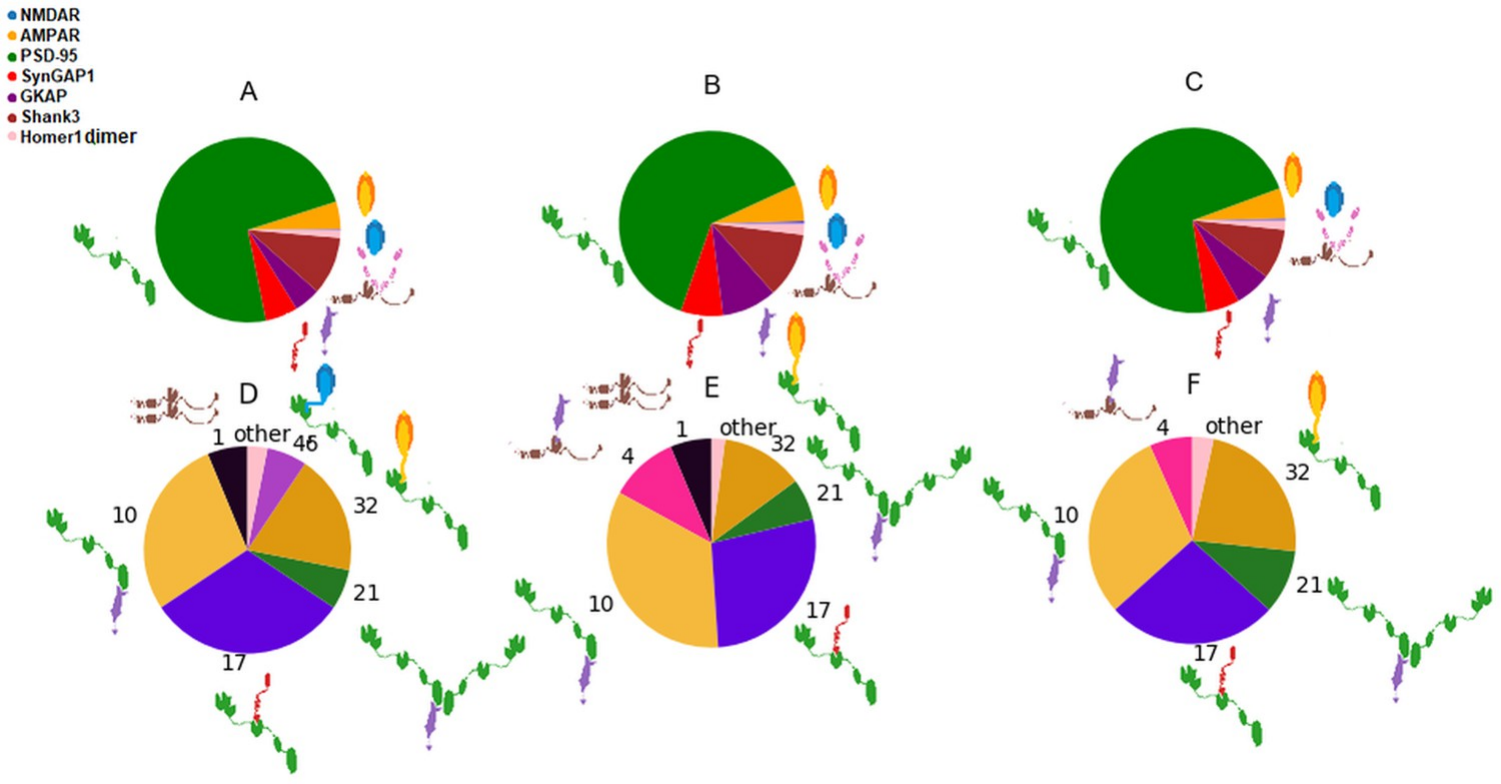
Comparison of input protein and output complex abundances for region 238 and the two regions with most similar input protein data. A, B and C: Input protein abundances in regions 238, 239 and 244, respectively. D, E and F: output complex abundances of regions 238, 239 and 244. The pie charts show the fractions of input proteins / output complexes. The complexes with abundances less than 2 are merged and shown as ‘other’. The charts demonstrate that relatively small changes in input protein abundance ratios can lead to substantial redistribution of complex fractions. Two of the complexes with the largest changes (Shank3 dimer, id 1; and Shank3/GKAP, id 4) do not contain the most abundant protein PSD-95 and their emergence does not even seem to be trivially dependent on the ratio of their constituent proteins.

We have identified the two regions with inputs (protein abundance data) most similar to region 238 and compared their respective outputs (complex distribution) with each other. Although the inputs are highly similar (see Fig 7), remarkable differences can be observed in the outputs, providing a clear example for the nontrivial relationships between protein and complex occurrence and demonstrating added information of the applied simulations.

All three regions are from the same individual, one sample from the ventrolateral prefrontal cortex (region 244) and another from the orbital frontal cortex (region 239). In these regions, the abundances of Homer1 and SynGAP1 show the largest difference compared to region 238. However, the most evident difference between the regions is in the presence of Shank3 dimers which are absent from regions 244. Instead, Shank3 proteins participate in several larger complexes or in the binary complex GKAP/Shank3. We can clearly see that a slightly less GKAP does not result in higher Shank3 dimer ratios, indicating a nontrivial relationship between input ptrotein abundance and complex formation in our simulations.

#### Regions 254 and 339: Different outputs from similar input abundances

Experiment 254, corresponding to the primary visual cortex (H376.IX.52_V1C) and 339 from the primary sensory cortex (H376.VIII.51_S1C) exhibit the second and third largest all-against-all distance differences between their input and output data vectors. Interestingly, these two sets have only slightly different input protein abundances but are not the closest sets of each other in this respect. The two inputs differ in the ratios of proteins SynGAP1, GKAP and AMPAR. The main difference between the sets 254 and 339 lies in their PSD-95/GKAP and SynGAP1/PSD-95 ratio as we can expect from the input differences. However, the SynGAP1/PSD-95/GKAP ternary complex can be expected to emerge in both simulations but less SynGAP1 and more GKAP does not lead to forming their ternary complex with PSD-95. In this case (experiment 254) higher ratio of the PSD-95(2)/GKAP precludes GKAP to join the SynGAP1/PSD-95 binary complex.

**Fig 8:**
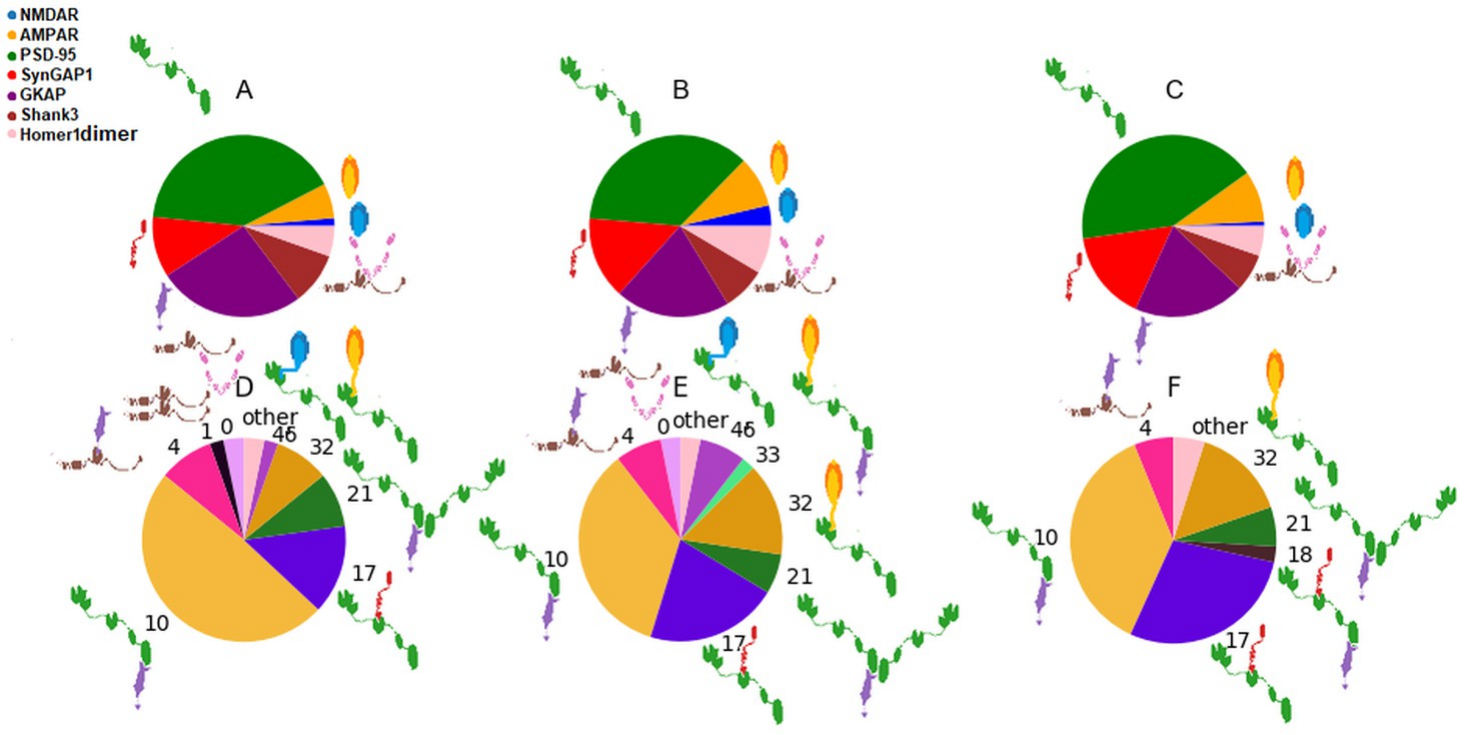
Input protein and output complex abundances for experiment 254 and the two sets with most similar inputs. A) Input protein abundances of region 254, B) input protein abundances of region 342, C) input protein abundances of region 255, D) output complex abundances of region 254, E) output complex abundances of region 342, F) output complex abundances of region 255.

For both regions, we have identified the experiments with highest similarity in the input abundances. For region 254 (see Fig 8, the complex distributions of the regions with most similar protein abundances are also highly similar to each other. The numerically largest differences can be observed of complexes 10 (PSD-95/GKAP)and 17 (SynGAP1/PSD-95). In the pie charts complex 1 (Shank3(2)) is seemingly missing from the other two regions and the ternary complex 18 (SynGAP1/PSD-95/GKAP) occurs only in the region 254 but their abundance is just below out threshold (2) for individual labeling.

**Fig 9:**
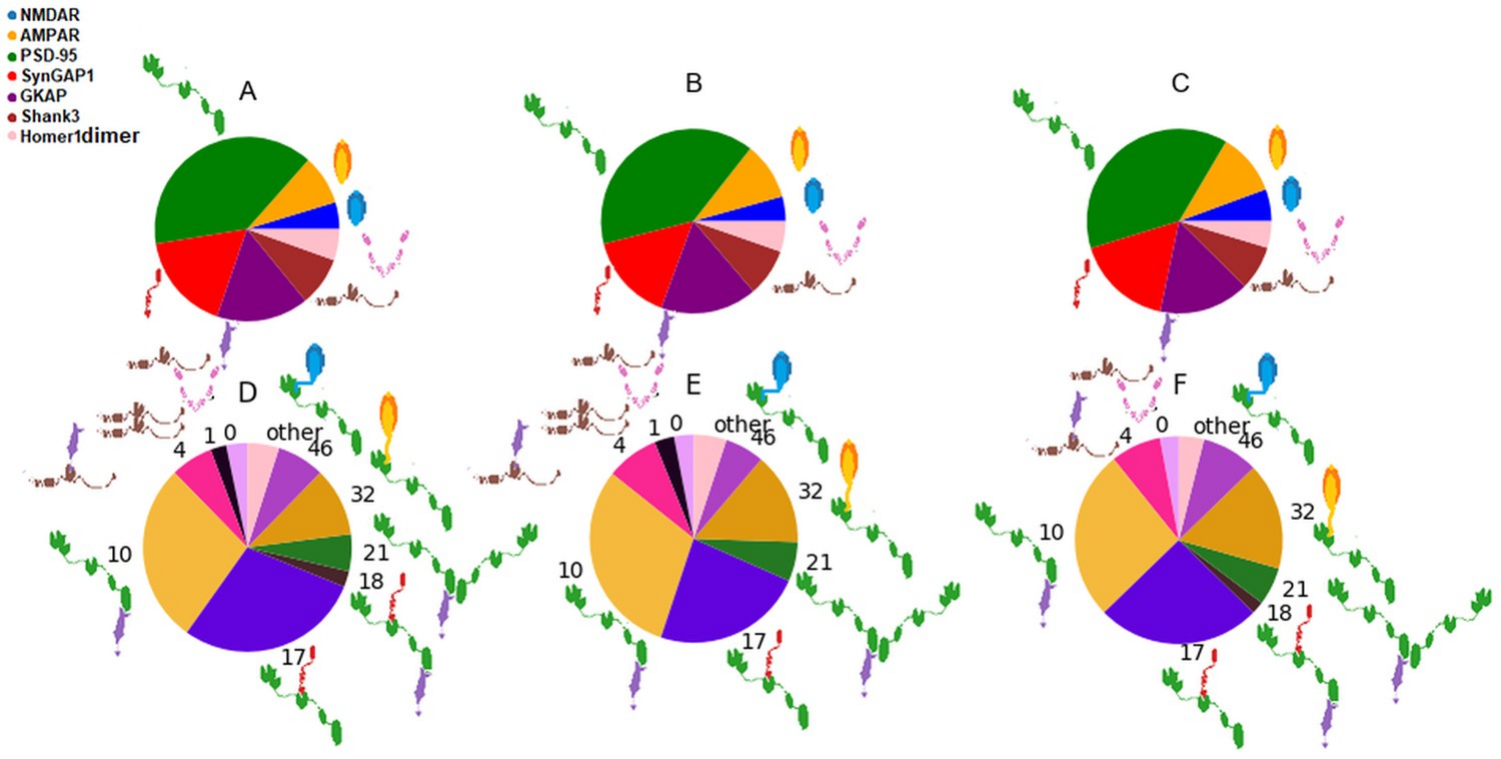
Input protein and output complex abundances for experiment 339 and the two sets with most similar inputs. A) Input protein abundances of region 339, B) input protein abundances of region 328, C) input protein abundances of region 340, D) output complex abundances of region 339, E) output complex abundances of region 328, F) output complex abundances of region 340.

Region 339 exhibits similar differences compared to its two closest regions in terms of protein abundance with also the largest differences in the abundance of complex 17 (SynGAP1/PSD-95) and 10 (PSD-95/GKAP) while having the largest ratio (Fig 9). Here, although the absolute abundance of complex 32 (AMPAR/PSD95) is highly similar (13, 14 and 17 complexes in region 339, 328 and 340, respectively), its relative ratio within all complexes can be different.

### Effect on simulation time on the evolution of complexes

Our results are dominated by relatively small complexes of 2–4 proteins. However, the potential interactions between the molecules allow for the formation of much larger associations, like complex 88 that contains four different kinds of proteins including seven copies of Shank3 see Fig 2. To explore the potential evolution of smaller complexes to associate and form larger ones, we have checked how large complexes evolve during the course of our simulations. We note that due to the nature of the Gillespie algorithm implemented in Cytocast, simulation time does not linearly scale with the number of steps. For each step, the algorithm calculates how long it takes to complete the next reaction [38]. For this reason, the relationship between simulation time and number of steps is not exactly linear, but shows noisier characteristics. As a result, duplicated simulation time does not necessarily mean exactly duplicated number of steps. We have analyzed our simulations at one-sixth and one-third of the full simulation time. We found that the most abundant complexes are already formed at one-sixth of the simulation time (t = 1/6). This observation indicates that the simulations reach an almost steady state already in relatively early stages, thus, the observed output is largely robust with respect to simulation time. Nevertheless, both the maximum and average size of complexes generally increases with time, indicating the presence of a slow association process leading to the emergence of supercomplexes.

The supercomplexes formed are typically unique and are present in very low copy numbers, usually 1. It seems still true that the number and distribution of the most abundant smaller associations does not change significantly, although the proteins are in constant dynamic interchange between the complexes. The weighted average of complex size remains in the range of 2.7–2.8 irrespective of the simulation time, meaning that binary and ternary complexes remain the most abundant.

Comparing the complex abundances of regions 238, 239 and 244 with the original simulation time and t = 1/6 (of the original time) demonstrates that the percentages of the complexes remain similar throughout the full simulation (Fig 7).

Principal component analysis of the outputs including the longer simulations for the three selected regions (238,254,339) corroborates the above observation that simulation time has negligible effect on the complex abundances, especially when compared to the changes observed for variations in the input protein distributions.

Notwithstanding the fact that the most abundant complexes remain the same when running the simulations six times longer, the maximum size of appearing complexes differ. The largest complex at t = 1/6 contained 11 proteins, the one at t = 1/2 had 8 and for the full simulation, 12 proteins throughout every region. Meanwhile, the average complex size remained 2 proteins.

Longer simulation time does not change the occurrence of the most abundant complexes, that are in fact much smaller than the large ones with very low abundance. The small, abundant complexes can be regarded as building blocks for the larger ones formed via their association. Thus, the simulation time affects the largest complexes in a way that the longer the simulation, the more small complexes stack together into supercomplexes without changing the dynamical equilibrium of the smaller blocks in the system.

Tracking the formation of large complexes is a nontrivial task as identification of the complexes during assembly and disassembly can not be easily solved. Therefore, our current implementation does not contain such a feature that would allow such analysis. Nevertheless, analysis of selected runs at a higher time resolution strongly suggests a scenario where a number of smaller complexes associate to form larger ones instead of each complex growing gradually by the addition of single components.

### Effect of higher-order associations of Homer1 and Shank proteins

Our simulations were ran in three variants, the most realistic one termed H4SM—discussed so far—considering Homer1 tetramerization via its C-terminal coiled coil region and Shank3 multimerization through its SAM domain, H4 considering only the former and also a variant where neither of these higher-order associations were included. Homer1 can form a dimer of dimers, and in the Simple simulation only dimers were considered, still capable of forming bivalent interactions *via* the EVH1 domain of each monomer.

In our H4SM simulations, the largest complex observed contains 12 proteins. Primary complexes typically associate through Shank3 multimerization with the occasional involvement of Homer1 tetramers. The supercomplex containing the most receptor molecules (2) observed during our simulations is unexpectedly held together only by one GKAP molecule. Receptors are not present in the largest complexes generated by Shank3 multimer chains.

**Fig 10:**
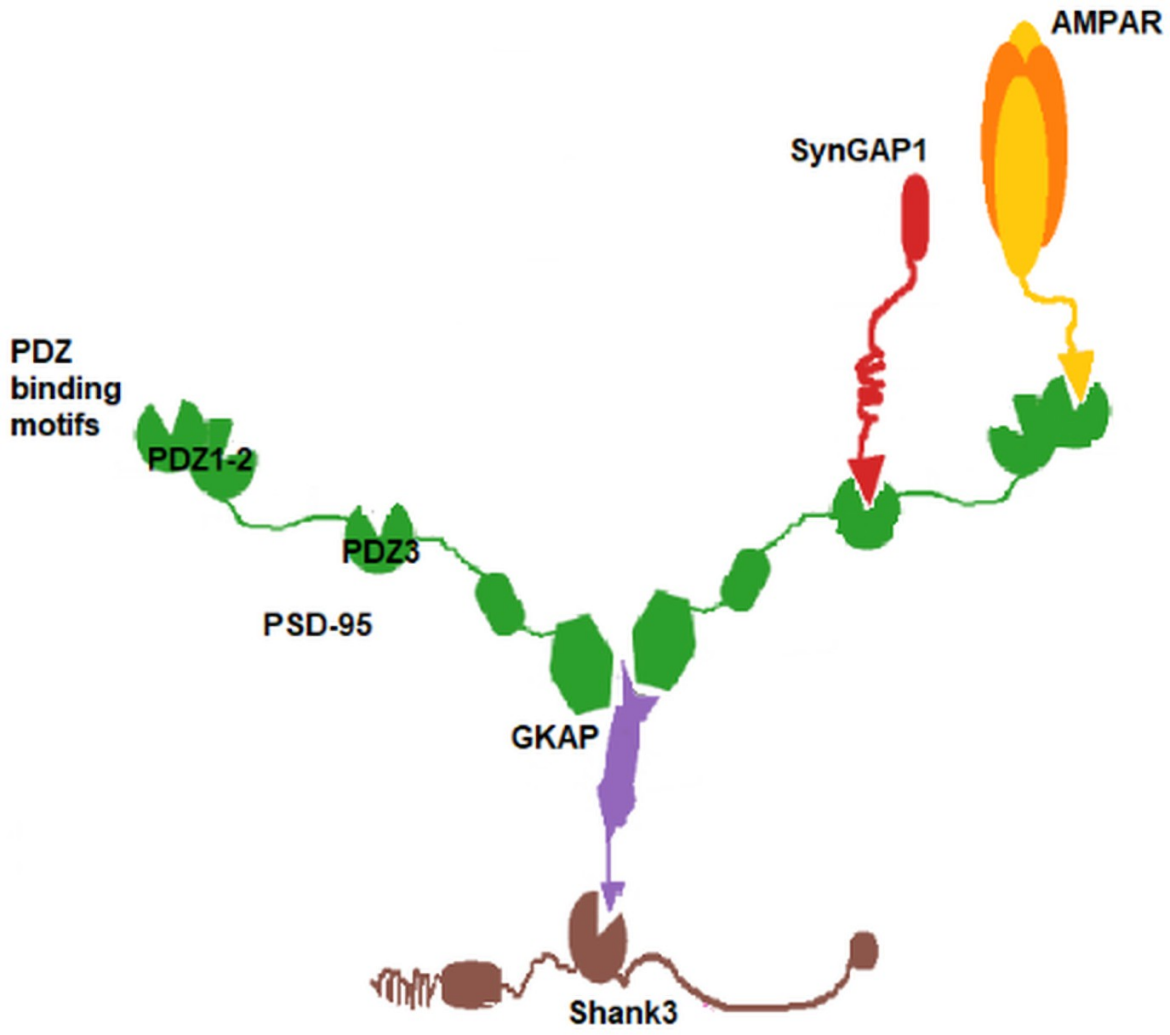
The structure of the largest complex created by Homer1 tetramerization. Homer1 is missing in the complex due to small number of Homer1 dimers in the system.

**Fig 11:**
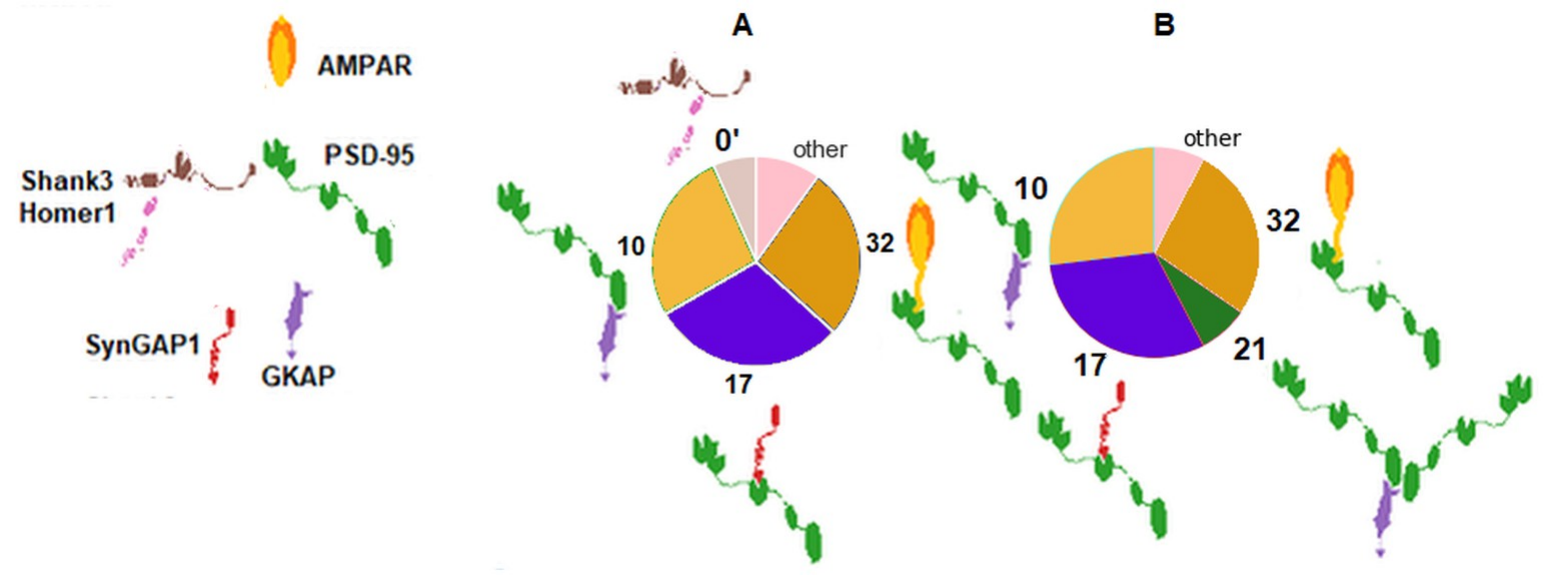
Distribution of complexes obtained for the region 238 with setups without SAM domain-mediated Shank multimerization. A) Simple setup (Homner dimers only, no tetramers) B) H4 setup (Homer tetramers allowed). The main complexes remained the same as in the setup H4SM (with both Homer tetramerization and Shank multimerizaton present).

Omission of Shank multimerization in our H4 simulations precludes the formation of very large supercomplexes. The largest complex observed in the H4 simulations is shown in Fig 10. This complex does not contain Homer1. There was no Homer1 tetramerization throughout these simulations. Homer1 was observed in three different complexes, mostly in the Shank3/Homer1(2). Since we consider every Homer1 molecule as a dimer, their low number means a small probability for tetramerization. Several similar complexes were also observed with one or two of the constituent proteins missing. In our Simple simulations, the relative abundances of primary complexes was similar to those in the H4 and H4SM simulations (Fig 11) but no large supercomplexes were formed. In general, Shank3 multimerization and and Homer1 tetramerization increased the diversity of the obtained complexes predominantly *via* the association of primary ones.

### Effect of variations in binding-unbinding rates

In the analysis throughout this paper, we used nominal binding—unbinding rates for each reaction. We made this simplification as there is no data on measurements. Although equilibrium dissociation constants (kDs) of the simulated interactions can be measured in-vitro and some (sometimes conflicting) data is available on these (Table L in S1 Table), but under *in vivo* conditions most often the abundance of binding partners is the rate limiting factor [39, 40].

To test the effects of changes in binding-unbinding rates in our simulations, we ran simulations with non-uniform binding values taken from the literature (Table L in S1 Table). Although some of these rates differ from the originally used nominal values, the results of these simulations show high similarity to the simulations with nominal binding rates (Fig F in S1 Text). However, the altered unbinding values can affect the association rate of larger complexes due to the lower unbinding probability some complexes that remain intact for a longer time and thus can stick together with a higher probability.

This observation highlights that indeed the abundances of binding partners are key to determine the abundances of their protein complexes, but the highly challenging measurement of their *in vivo* binding kinetics might not be that crucial.

## Discussion

### Complex distributions and synaptic identity

The synaptomic theory, formulated by Seth Grant [2] proposes that the diversity of synapses in terms of the protein complexes and supercomplexes is key in governing neural functions. In this respect, investigations of the protein complexes and their relationship with protein abundance is indispensable to get closer to the understanding this variability and its genetic regulation.

The main observation in our simulations is that the distributions of the emerging complexes are in an intricate relationship with the abundance of their constituent proteins. Because of the many possible interactions, the availability of given proteins to form a certain complex depends on all of their potential partners, providing considerable interdependence between the abundance of different protein associations. While higher abundance of a partner protein might mean higher chance of binding in simple cases, in other scenarios this abundance might lead to the sequestration of the abundant partner into many different complexes depending on the availability of its additional partners. This kind of interdependence can only be quantitatively assessed by simulations like those presented in our work. Our simulations show that even small changes in protein distributions can lead to a remarkable redistribution of complexes even in a simplified model of the PSD. Our observations reveal that protein abundance alone can be a major organizer of PSD structure even when posttranslational modifications and other factors potentially influencing the availability and of binding sites and the strengths of interactions are not considered. The presented examples of regions with similar protein but more divergent complex abundances suggest that local synthesis and degradation of selected proteins can lead to the redistribution of protein complexes at a degree that can remarkably change the ‘identity’ of a synapse. The presence of local mRNA translation in dendritic spines, producing, among others, PSD proteins, is well established and has been associated with a number of neuronal processes like late-phase LTP [41]. Also, similar complex distributions might be achieved with different sets of the constituent proteins, providing the potential of multiple ways of achieving functionally similar states. Our observation is that neither PCA nor tSNE analysis shows clear grouping of data sets from the same brain region and/or individual. This suggests that the variation observed can not be easily related to these aspects.

### Protein supercomplexes, nanodomains and the organization of the PSD

Our simulations suggest that Homer1 tetramerization alone is not enough to make the formation of large supercomplexes possible, as these supercomplexes are only emerging when Shank3 self-association is also introduced into the model. The observation that the abundance of primary complexes do not exhibit large variations in the different settings suggests that the organization of supercomplexes is hierarchical, they can be formed from and broken down into smaller associations. The presence of several large supercomplexes is qualitatively compatible with observations indicating the presence of functional “nanodomains” in the PSD [42, 43]. The dynamic reorganization of large complexes *via* the dissociation and association of smaller associates is probably a key mechanism and is also in line with the observations pointing to changes in the composition and size of the PSD [44]. Our observation that the average and maximum size of the complexes incrseases with simulation time indicates that technically we might reach an equilibrium over a very long simulation time. However, the information content of such a steady state might be low because if the majority of the proteins are found in only a few very large associations, the diversity of the complexes would be low and as such large complexes would be unique making comparisons between simulations difficult. In the extreme case all the proteins might form a single supercomplex which is both unrealistic and uninformative as the complex composition would eventually reproduce the input abundances. Such a scenario might be prevented by introducing more realistic association-dissociation constants and/or protein turnover into the simulations. Our calculations are robust, the complex distributions do not show large fluctuations during the course of the simulations, rather adopt a quasi-equilibrium relatively quickly. If the simulation outputs were largely dependent on stochastic fluctuations, then repeated calculations would also show large random divergence, which is not observed. In contrast, the sensitivity of the results to specific alterations of the input abundances is well reproduced. Notably, small variations in the input abundances cause deviations in the output complex distributions in only a number of well-defined cases.

### Models of PSD complexes—How far are we from reality?

Protein copy numbers estimated from linear scaling of mRNA expression data do not take into account translational and posttranslational regulatory effects. Such effects may result in greater variance of complexes between brain regions. Ideally, direct protein abundance data would be needed to get more realistic simulation results. Imaging techniques are available to get closer information about protein abundances. These imaging techniques show similar proportions to the mRNA data as validation but the dimensions are not exact protein numbers but voxels and intensities [45], and such data are much more sparsely available than mRNA expression data. The detailed description of PSD organization is still a challenge. It is complicated by its size, the number and variations of constituent proteins and, most of all, its variable stoichiometry and dynamics. Although high-resolution experimental data on binary complexes are available, these typically only contain the interacting domains and segments. The full PSD can be isolated but from it only the abundance of the constituent proteins can be estimated by mass spectrometry [46]. To our best knowledge, even in vitro reconstructions of PSD complexes do not provide information at the level that could be directly comparable to the results of our simulations [5].

Our premise is that simulations can complement experimental data and can meaningfully contribute to our understanding of the nature of the PSD. The modeling approach presented here is a first-approximation one focusing on the variability of protein abundances and only a highly simplified set of seven PSD proteins and only a single variant of each. Thus, its complexity is far from the actual organization of the PSD. Consequently, our results might not have direct relevance to the actual distribution of complexes in the PSD in different neurons. The ideal case would be to simulate the entire PSD (or a significant portion of it) using experimentally determined binding and unbinding rates. Currently, we are very far from this scenario and these two aspects mutually exclude each other. Even for the 7-protein system investigated there are very few *k*_*on*_ and *k*_*off*_ rates, only *K*_*d*_ values have been determined and even these vary between different experiments performed by various research groups. Expanding the protein set would mean using many more unknown binding rates. Availability of a larger amount of quantitative experimental data, especially on complexes, would also make it possible to perform detailed studies related to model identification in terms or protein-complex abundance relationships similar to the one described in [47]. Alteration of the binding and unbinding rates can show how the results are dependent on the altered rates. More precise simulations would require quantitative data on abundances directly at the protein level, appropriate binding constants and the consideration of the 3D organization of the complexes together with their localization within the postsynaptic region. In addition, dynamic turnover of the components, the spatial direction of the addition of new proteins as well as the phenomenon of phase separation are all issues that are expected to contribute to the actual distribution of the complexes *in vivo*. We believe that in the future simulations, following the proposed method, will be able to provide information for experimental design to test the abundance of specific large complexes or interactions, making use of the continuously developing molecular imaging techniques.

## Materials and methods

### Data sets

Data described in [12] has been processed by an in-house Python script. The source data was stored in three CSV files where one contained the protein abundance data in a matrix while the other two contained the information of the columns and rows.

The data set contained RNA-Seq RPKM (reads per kilobase per million) values averaged to genes. From these values protein abundance data were generated as below:

1. The median RNA-Seq value of the PSD-95 were calculated.
2. This value (66.82 rpkm) were assigned to the protein abundance 300 [48]
3. RNA-Seq values were multiplied by 300/66.82—assuming linear relationship based on [49]

The model is suitable for making a primary estimate. Approximating the protein abundances by mRNA expression shows interregional separation at the level of mRNA synthesis, while other separations could emerge during the translation of the proteins. In the case of actual protein abundance data, the procedure can be repeated in the same way without changes. The distribution of the data is not uniform in the sense that there are brain regions for which data are not available from all of the patients. Because of this we handled all experiments separately and aimed at identifying the major differences between the results.

### Simulations of complex formation

Cytocast implements a version of the Gillespie algorithm, which is a Monte Carlo based method to simulate every reaction in order to have a non-deterministic approach of abundance change of molecules or in our case complexes.

Steps of Gillespie-algorithm [50]:

1. Set the initial conditions as the number of starting molecules (here protein abundance) *n*_*i*,0_ and possible reaction numbers *q*
2. Generating two random variables between 0 and 1. (*r*_1_, *r*_2_)
3. Compute the propensity functions of each reaction (here binding, note if a binding occurs there is one less protein).
  $$\begin{array}{c} \alpha_{i}\left(t\right)=\left(n_{i,t-1}-1\right)k_{i} \end{array}$$ (1)
4. Compute the propensity function for the whole system:
  $$\begin{array}{c} \alpha_{0}=\sum_{j=1}^{q}\alpha_{j}\left(t\right) \end{array}$$ (2)
5. Compute the time when the next chemical reaction takes place as *t* + *τ* where
  $$\begin{array}{c} \tau =\frac{1}{\alpha_{0}}\text{ln}(\frac{1}{r_{1}}) \end{array}$$ (3)
6. Calculate which reaction occurs then adjust the numbers of the proteins (one less protein from the inputs and one more for the output (complex)). The *i*^*th*^ reaction occurs if the conditions are true:
  $$\begin{array}{c} \frac{1}{\alpha_{0}}\sum_{j=0}^{i-1}\alpha_{j}\leq r_{2}<\frac{1}{\alpha_{0}}\sum_{j=0}^{i}\alpha_{j} \end{array}$$ (4)

The equilibrium dissociation constant (*K*_*D*_) is the ratio of unbinding (*k*_*off*_) to binding rate (*k*_*on*_).

**Cytocast** is a Gillespie-based stochastic modeling software of agent-based protein-binding in a virtual cell where the proteins are point like. The software gives a quantitative prediction on complex abundances with certain initial conditions. These conditions are the simulation time, compartments’ size and shape, protein abundances, diffusion rates, protein functions and bindings. The software was based on the publicly available SiCompre [19].

As it is unrealistic to have binding and unbinding rates for all possible reactions in the system, we set these rates uniformly to 1 a.u. (binding = 1 a.u., unbinding = 1 a.u.). Although the ratio of the rates affects the size of the emerging complexes, our intention is to investigate the effect of varying protein abundances, which in this case will be the main determinant of the results. Previous works have shown that this simplification can lead to biologically relevant results [14, 19].

### The compartment used for the simulations

The compartment was set up in 3 dimensions and had a spherical shape in order to approach the shape of a general dendrite. The sphere contained 1024 subvolumes. This shape and size gives enough space for the proteins to diffuse but small enough to observe sufficient number of interactions.

### Online tool for simulations

The online tool (http://psdcomplexsim.cytocast.com/) calculates protein complexes of the seven major postsynaptic proteins (two membrane receptors, NMDAR and AMPAR, as well as five scaffold proteins) as a function of their individual abundances. The abundance of the constituent proteins can be set to any desired value using the form. By default, Homer proteins are modeled as dimers and Shank polymerization (via its SAM domain) is not considered, you can change these settings by ticking the appropriate boxes. The default length of the simulation run with this service is 1 AU. The output contains all the protein complexes formed from the user set abundances of each proteins. The tool allows the reproduction of the results described in the present paper and enables users to test any combination of protein abundances.

### Analysis of the simulations

#### Dimension reduction methods

Two main dimension reduction were used analyzing the data. Principal Component Analyses is based on the eigenvectors and eigenvalues of the data-set. The calculated eigenvectors are the principal components and their corresponding eigenvalues show the contribution of those vectors to the variance of the dataset. During the analyses we transform the original coordinates into the base of eigenvectors. The coordinates of the eigenvectors are related to the weight of the original axes in the given eigenvector.

For input data seven dimensions should be reduced:

$$\begin{array}{c} v=a_{1}p_{1}+a_{2}p_{2}+\cdots +a_{7}p_{7}=\alpha_{1}s_{1}+\alpha_{2}s_{2}+\underset{\text{axis}\;\text{not}\;\text{shown}}{\underset{︸}{\cdots +\alpha_{7}s_{7}}} \end{array}$$ (5)

In the Eq 5 a data set is represented by the input protein abundances. The *a*_*i*_ is the abundance of the protein *p*_*i*_. The *p*_*i*_ is the axes representing the protein *i*. On the other side of the equation there are the new coordinates of the data set *α*_*i*_ where *s*_*i*_ is the eigenvector *i*. The first eigenvector represents the largest contribution (eigenvalue) and the last one has the lowest impact. The calculations were made by scikit-learn [37].

Another dimension reduction method is the t-distributed Stochastic Neighbor Embedding (tSNE), which is based mainly on joint probabilities created by the dataset. tSNE converts Eucledian distances into Gaussian probabilities where the probability is high if the two points are close.

$$\begin{array}{c} P\left(v_{j}|v_{i}\right)=\frac{e^{\frac{-\left|\right|v_{i}-v_{j}{\left|\right|}^{2}}{2\sigma_{i}^{2}}}}{\sum_{k\neq i}e^{\frac{-\left|\right|v_{i}-v_{k}{\left|\right|}^{2}}{2\sigma_{i}^{2}}}} \end{array}$$ (6)

Calculating the joint probabilities:

$$\begin{array}{c} P\left(v_{i},v_{j}\right)=\frac{P\left(v_{i}|v_{j}\right)+P\left(v_{j}|v_{i}\right)}{2n} \end{array}$$ (7)

For low-dimensional spaces, a Student-t distribution is calculated. Using the two probabilities an error function can be defined which is then minimized by an optimization approach [51]. We used the scikit-learn for running the algorithm.

#### K-means clustering of one-hot labeled regions

The K-means clustering is one of the simplest algorithms for creating similarity classes so called clusters based on closeness in a parameter space. The number of clusters is a key parameter as the same difference can be seemingly larger when a higher number of clusters is used. In this study we have performed K-means clusterings for K = 2,3,4,5 and 6 (see Fig A in S1 Text).

The optimal cluster numbers were chosen by visualizing silhouette scores of clustered data both on input field (Fig B in S1 Text) and on output field (Fig C in S1 Text) for each K.

Clusters were formed both from the input and output data. The inputs were considered vectors in a seven dimensional vectorspace R^7^. Each dimension was assigned by a protein abundance. The outputs were represented as a six dimensional one R^6^ where each dimension describes the abundance of a selected interaction.

The algorithm in short (Python packages and MatLab have their own implementations) [52]:

1. Randomize the *k* mean points.
2. Each data point *n* is assigned to the nearest mean.
3. Calculate the new mean of the clusters by the assigned data points.
4. Reassign each data point to the new nearest mean.
5. Repeat step 3 and 4 until every mean point remains unchanged.

Then we generated a one-hot labeled vector for each cluster. The coordinates of the vector represent each dataset, and the coordinate is 0 if the dataset is not in the cluster and one if the data is in the cluster.

$$\begin{array}{c} c\in {\left\{0,1\right\}}^{\text{Number}\;\text{of}\;\text{Data}\;\text{Points}} \end{array}$$ (8)

$$\begin{array}{c} c_{i}=\{\begin{array}{c} 1\;\text{if}\;{\text{data}}_{i}\in Cluster\\ 0\;\text{if}\;{\text{data}}_{i}\notin Cluster \end{array} \end{array}$$ (9)

Then the distance of two clusters can be formalized as:

$$\begin{array}{c} d\left(u,v\right)=\frac{\sum_{i=1}^{N}|u_{i}-v_{i}|}{N} \end{array}$$ (10)

Where *u,v* are cluster vectors and *N* is the number of datapoints (dimension of the cluster vectorspace). This is a conservative measure in the sense that points that are not included in any of the two clusters are also considered to be similar. For this reason, even smaller values represent a larger difference compared to considering only the union of the two clusters.

High inter-cluster distances indicate that the input and the output data are in a nontrivial relationship.

#### Differences between region-to-region similarities based on protein and complex abundances

The brain regions were associated by a point in a multidimensional space, one space for the input data and another space for the output data. For the input data the axis were the protein abundances while in the output data the axis were the occurred complexes. Eucledian distances between each brain regions were calculated in the input data and in the output data as well. Then those distances were normalised between zero and one to eliminate artefacts caused by the different vector spaces before checking how a distance changed. The two distance matrices were then subtracted to identify the largest changes in region-to-region similarities based on protein and complex abundances.

#### Visualization of protein complex distributions

The real question of simulations, however, is what complexes appear in a given simulation and in what quantities. It is difficult to compare two simulations based only by the specific numbers of complexes, since our starting materials the proteins, are also present in less or more amounts. Thus, for the comparison, the relative abundances of the complexes were taken into account. The pie charts show that a given complex occurs at what percentage of the whole complexes. The complexes with the smallest percentages were put together in the other group by a threshold of amount. Thus,the complexes which were the most common according to the simulation in the given brain region can be determined. The complex IDs are generated for one simulation set containing all the regions to be compared. Therefore, when comparing different metaparameters on same regions, the complex IDs emerged have to be associated with the original IDs of the same complex, in the charts the colors are also associated with the original IDs.

## Supporting information

S1 Text **Fig A**. **Analysis of the relationship between the input and output data based on cluster distances obtained for different cluster numbers generated by K-means clustering**. **Fig B**. **Silhouette scores of kMeans algorithm on input data**. The red line indicates the average score. Higher scores indicate more reliable clustering. Each cluster is colored differently. Although the average score is highest at k = 2 for the output complexes, the smaller cluster is virtually non-existent at this value. Thus, practically the the k = 3 and k = 4 cases can be used for informative comparisons. **Fig C**. **Silhouette scores of kMeans algorithm on output data**. **Fig D**. **Correlations of Relative Positions of PCA points**. The heatmap shows how much the direction of the relative positions between each brain region differs from input positions to output positions regarding to their first two principal components. The value is absolute. It is illustrated at the bottom of the figure that the cosine of the intervening angle of the positions was taken. X is a reference point in the input and output field of while the A is an another point in the input field and A’ is the same region as A just in the output field. **Fig E**. **The distribution of data for each brain region along first principal component**. **Fig F**. **Distribution of complexes obtained for the three main regions with setup of non-uniform binding values and the two closest—in case of input protein abundances—regions for each (similarly to the main figures)**: A,D) H376.IX.51_MFC G,J) H376.VI.50_V1C M,P) H376.VIII.51_S1C. Similar to the region H376.IX.51_MFC are the regions: B,E: 239 and C,F:244. Similar to the region H376.VI.50_V1C are the regions: H,K:342 and I,L:255. Similar to the region H376.VIII.51_S1C are the regions: N,Q:328 and O,R:340. The regions with similar protein abundances (lines) remained similar however small perturbations still caused small changes as we observed originally. Changes between the uniform and non-uniform unbinding values makes differences between the complex abundances. The most abundant complexes remain the most abundant but the ratio changed and the less abundant complexes changed indicating that non-uniform binding rates affect more the time a bigger complex needs to evolve.For example on subfigure B there is the complex GKAP/Shank3 while on subfigure E for non-uniform unbinding values instead of the binary complex GKAP/Shank3 there is the quaternary complex of PSD-95(2)/GKAP/Shank3. This change is caused by the smaller (*k*_*D*_ = 0.02*μM*) unbinding value of GKAP-Shank3 binding. Similar phenomenon can be observed at the subfigures M-P and N-Q where the PSD-95/GKAP/Shank3 and PSD-95(2)/GKAP/Shank3 complexes emerged due to the smaller unbinding value of PSD-95-GKAP binding.
(PDF)

S1 Table **Table A**. **Input Proteins with Uniprot ID and Domains**. **Table B**. **Domain-Domain Interactions**. **Table C**. **ID of Brain Regions**. **Table D**. **Function of Brain Regions**. **Table E**. **Input protein abundances**. **Table F**. **Protein complex IDs**. **Table G**. **Output of H4SM Original** (rows:complexes columns:regions). **Table H**. **Output of H4SM t = 1/2**. **Table I**. **Output of H4SM t = 1/6**. **Table J**. **input PCA coordinates** **Table K**. **output PCA coordinates**. **Table L**. **equilibrium dissociation constants found in literature**.
(XLSX)

S1 Dataset Raw outputs of regions. The averaged and sorted outputs of each region of the corresponding simulation. H4: Homer tetramerization, SM: Shank3 polymerization and t time.
(ZIP)
