# Supplementary material for: Diversity of synaptic protein complexes as a function of the abundance of their constituent proteins: A modeling approach
Source: PLoS Comput Biol. 2022 Jan 18;18(1):e1009758. doi: 10.1371/journal.pcbi.1009758 (PMC8797218; doi:10.1371/journal.pcbi.1009758)
Supplement: S1 Text — Fig A. Analysis of the relationship between the input and output data based on cluster distances obtained for different cluster numbers generated by K-means clustering. Fig B. Silhouette scores of kMeans algorithm on input data. The red line indicates the average score. Higher scores indicate more reliable clustering. Each cluster is colored differently. Although the average score is highest at k = 2 for the output complexes, the smaller cluster is virtually non-existent at this value. Thus, practically the the k = 3 and k = 4 cases can be used for informative comparisons. Fig C. Silhouette scores of kMeans algorithm on output data. Fig D. Correlations of Relative Positions of PCA points. The heatmap shows how much the direction of the relative positions between each brain region differs from input positions to output positions regarding to their first two principal components. The value is absolute. It is illustrated at the bottom of the figure that the cosine of the intervening angle of the positions was taken. X is a reference point in the input and output field of while the A is an another point in the input field and A’ is the same region as A just in the output field. Fig E. The distribution of data for each brain region along first principal component. Fig F. Distribution of complexes obtained for the three main regions with setup of non-uniform binding values and the two closest—in case of input protein abundances—regions for each (similarly to the main figures): A,D) H376.IX.51_MFC G,J) H376.VI.50_V1C M,P) H376.VIII.51_S1C. Similar to the region H376.IX.51_MFC are the regions: B,E: 239 and C,F:244. Similar to the region H376.VI.50_V1C are the regions: H,K:342 and I,L:255. Similar to the region H376.VIII.51_S1C are the regions: N,Q:328 and O,R:340. The regions with similar protein abundances (lines) remained similar however small perturbations still caused small changes as we observed originally. Changes between the uniform and non-uniform unbinding values m [file pcbi.1009758.s001.pdf]

Diversity of synaptic protein complexes as a function of the abundance of their constituent proteins: a modeling approach

Marcell Miski[1], Bence Márk Keömley-Horváth[1,2], Dorina Rákóczi Megyeriné[1], Attila Csikász-Nagy[1,2,3*], Zoltán Gáspári[1*]

1 Faculty of Information Technology and Bionics, Pázmány Péter Catholic University, Budapest, Hungary
2 Cytocast Ltd., Vecsés, Hungary
3 Randall Centre for Cell and Molecular Biophysics, King's College London, London, United Kingdom

Supporting information - Figures

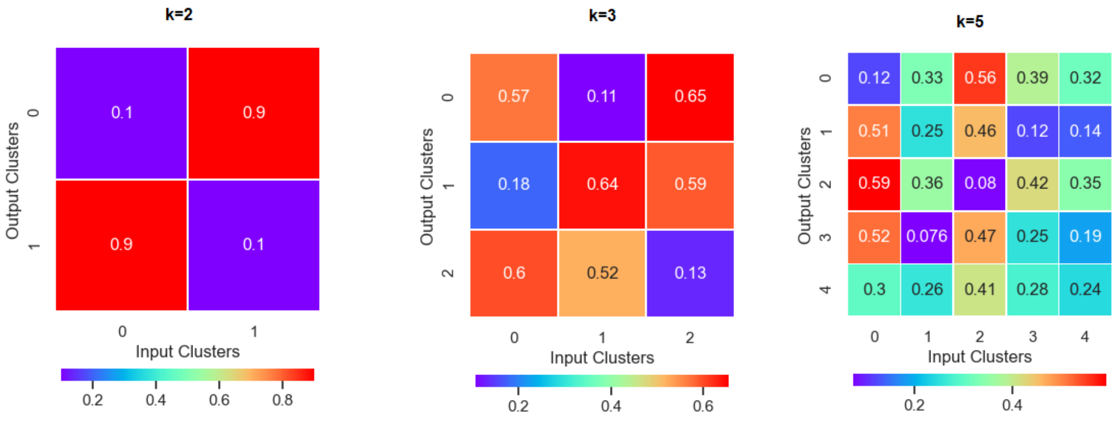

Fig A Analysis of the relationship between the input and output data based on cluster distances obtained for different cluster numbers generated by K-means clustering.

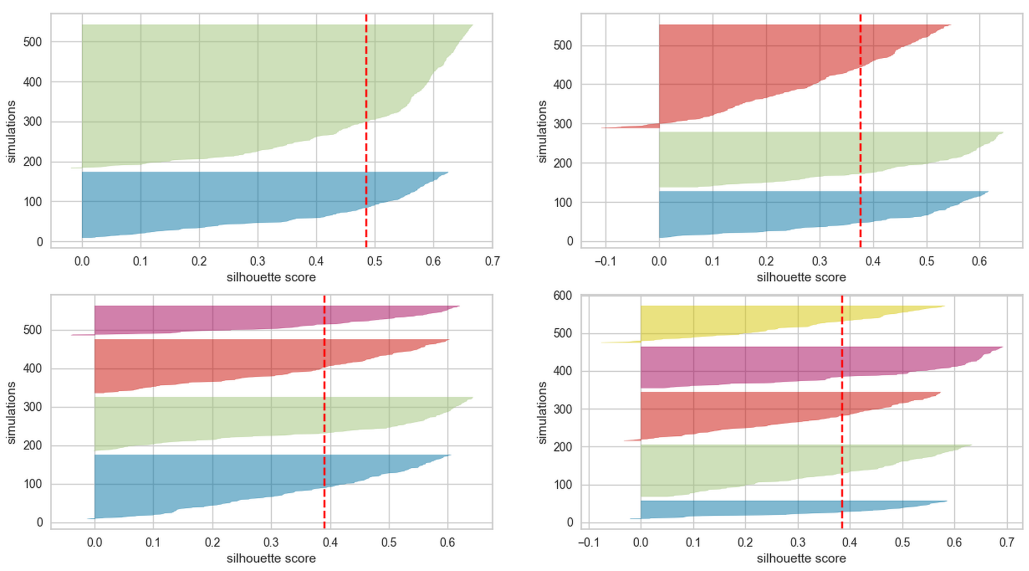

Fig B Silhouette scores of kMeans algorithm on input data The red line indicates the average score. Higher scores indicate more reliable clustering. Each cluster is colored differently. Although the average score is highest at k=2 for the output complexes, the smaller cluster is virtually non-existent at this value. Thus, practically the the k=3 and k=4 cases can be used for informative comparisons.

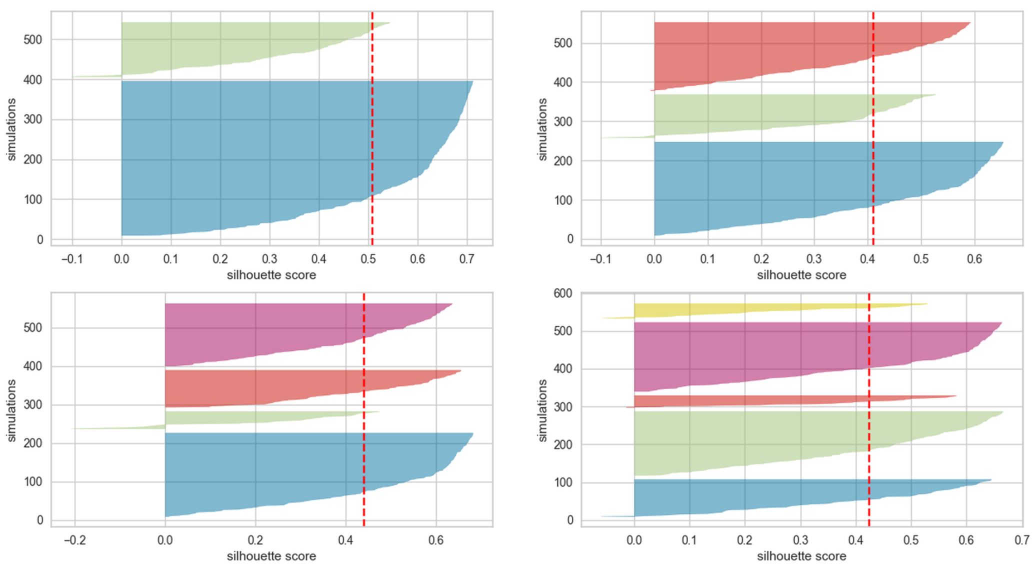

Fig C Silhouette scores of kMeans algorithm on output data

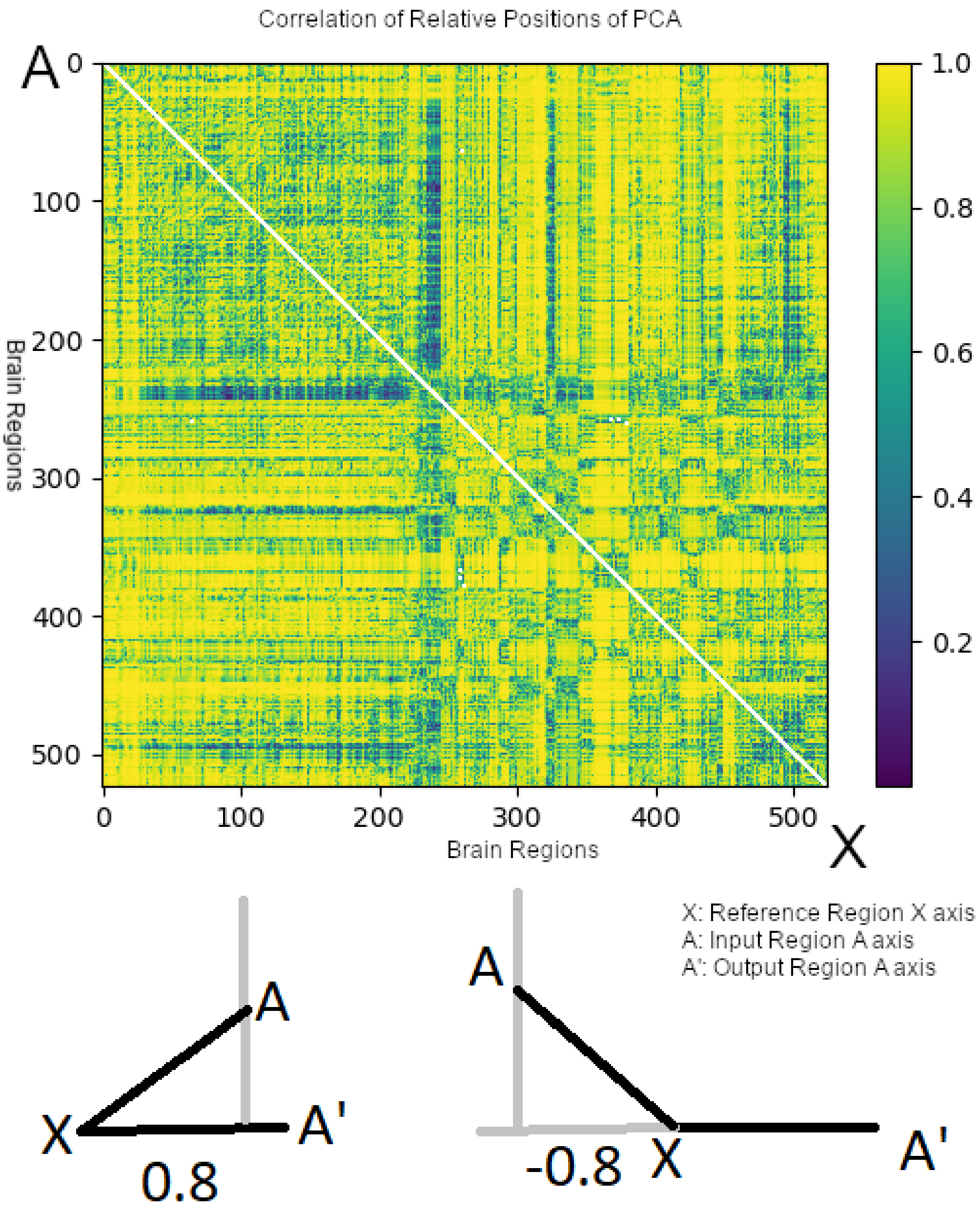

Fig D Correlations of Relative Positions of PCA points The heatmap shows how much the direction of the relative positions between each brain region differs from input positions to output positions regarding to their first two principal components. The value is absolute. It is illustrated at the bottom of the figure that the cosine of the intervening angle of the positions was taken. X is a reference point in the input and output field of while the A is an another point in the input field and A' is the same region as A just in the output field.

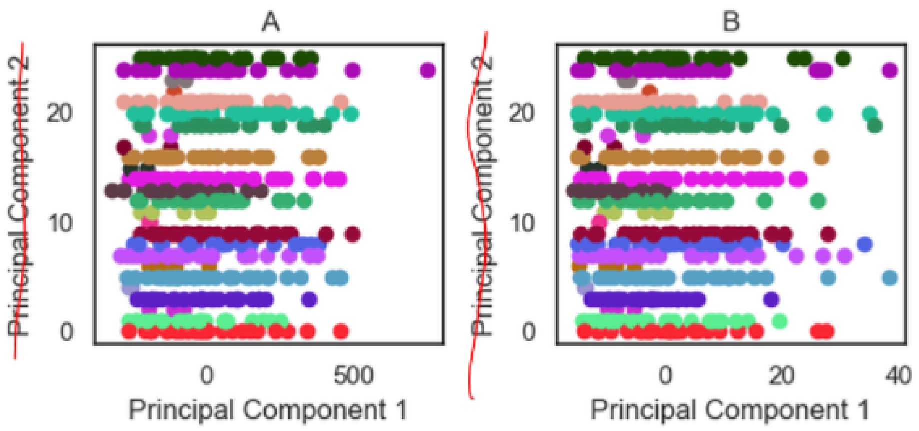

Fig E The distribution of data for each brain region along first principal component

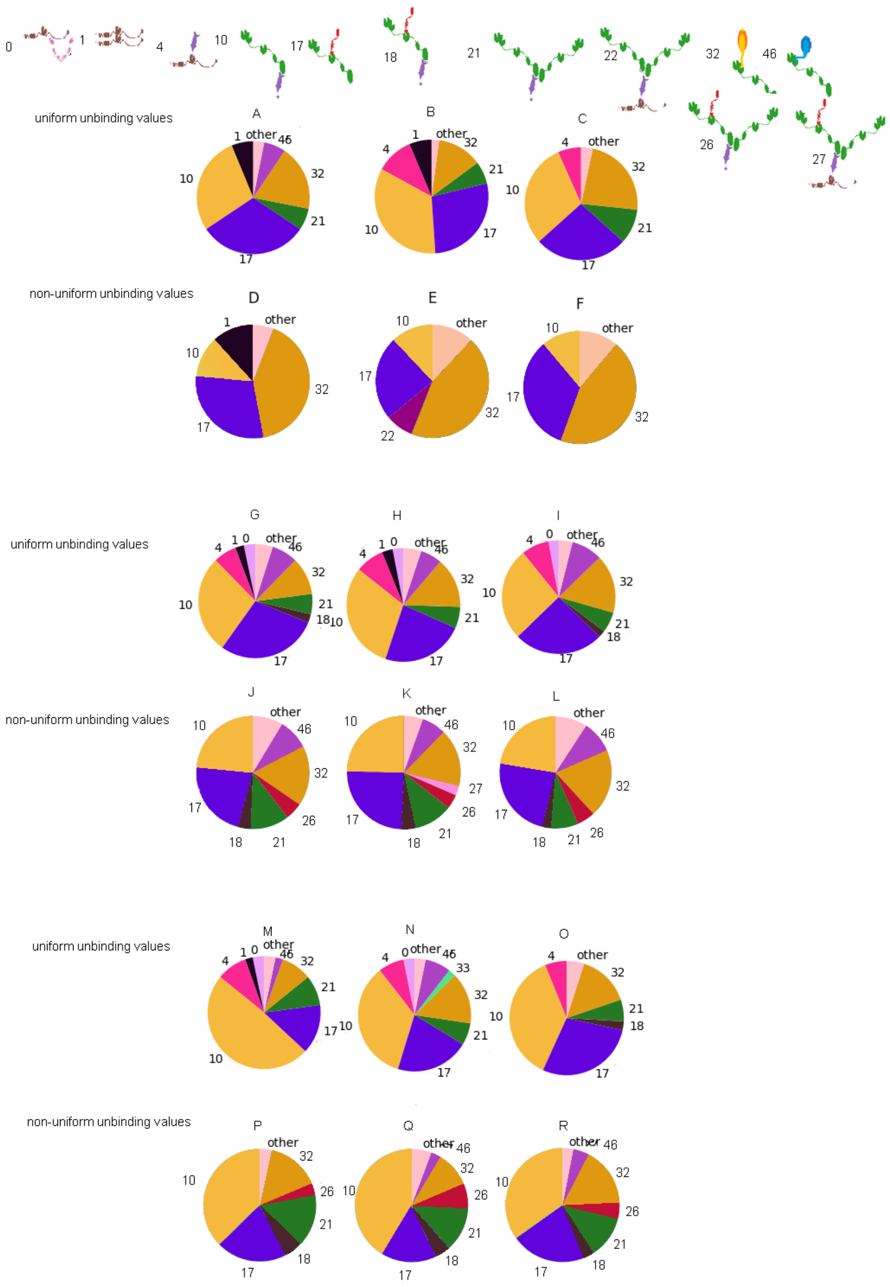

Fig F Distribution of complexes obtained for the three main regions with setup of non-uniform binding values and the two closest - in case of input protein abundances - regions for each (similarly to the main figures): A,D) H376.IX.51_MFC G,J) H376.VI.50_V1C M,P) H376.VIII.51_S1C. Similar to the region H376.IX.51_MFC are the regions: B,E: 239 and C,F:244. Similar to the region H376.VI.50_V1C are the regions: H,K:342 and I,L:255. Similar to the region H376.VIII.51_S1C are the regions: N,Q:328 and O,R:340. The regions with similar protein abundances (lines) remained similar however small perturbations still caused

small changes as we observed originally. Changes between the uniform and non-uniform unbinding values makes differences between the complex abundances. The most abundant complexes remain the most abundant but the ratio changed and the less abundant complexes changed indicating that non-uniform binding rates affect more the time a bigger complex needs to evolve.For example on subfigure B there is the complex GKAP/Shank3 while on subfigure E for non-uniform unbinding values instead of the binary complex GKAP/Shank3 there is the quaternary complex of PSD-95(2)/GKAP/Shank3. This change is caused by the smaller ($k_D = 0.02\mu M$) unbinding value of GKAP-Shank3 binding. Similar phenomenon can be observed at the subfigures M-P and N-Q where the PSD-95/GKAP/Shank3 and PSD-95(2)/GKAP/Shank3 complexes emerged due to the smaller unbinding value of PSD-95-GKAP binding. The unbinding values are available in the Supplementary Table 12.
